# Multiverse analysis of machine learning: classification between groups defined by suicidal ideation screening status using acoustic features in college students

**DOI:** 10.3389/fpsyg.2026.1785437

**Published:** 2026-06-29

**Authors:** Min Lyu, Lixin Tan, Fangjian Liu, Jingyu Lei, Tianxiang Jiang, Jiahui Qi, Xueqian Wang, Hui Yang, Jiang Zhong, Zhengzhi Feng

**Affiliations:** 1 Department of Medical Psychology, Army Medical University, Chongqing, China; 2 Mental Health Education and Counseling Center, Chongqing University, Chongqing, China; 3 Faculty of Health and Wellness, City University of Macau, Macau, China; 4 Department of Clinical Psychology, Chongqing Emergency Medical Center/The Fourth People's Hospital of Chongqing, Chongqing, China; 5 College of Computer Science, Chongqing University, Chongqing, China; 6 Department of Medical Psychology, First Affiliated Hospital of Army Medical University, Chongqing, China; 7 College of Medicine, Chongqing University, Chongqing, China

**Keywords:** acoustic features, college students, machine learning, multiverse analysis, suicidal ideation

## Abstract

**Background:**

Suicide is a major global public health crisis and a leading cause of unnatural death among college students. Current suicide ideation assessment mainly relies on self-report questionnaires and structured interviews. These methods are vulnerable to response bias and cannot support continuous monitoring. There is an urgent need for objective and non-invasive correlates of suicide ideation. Speech provides a promising source of such correlates, as acoustic features reflect emotional and cognitive states related to suicidal ideation. Although speech-based machine learning models have shown encouraging predictive performance, most studies rely on single analytical pipelines. Consequently, the robustness and generalizability of reported acoustic correlates across analytical choices remain a question for clinical translation.

**Methods:**

A comprehensive multiverse analysis was conducted across 1,764 distinct analytical pipelines using speech data from 96 Chinese university students (48 individuals who screened positive for suicidal ideation on the SIOSS and C-SSRS, and 48 matched controls who screened negative). The pipelines varied in preprocessing strategies, acoustic feature sets, dimensionality reduction methods, and machine learning models. Model performance was evaluated using the area under the receiver operating characteristic curve (AUC). Feature importance was aggregated across all pipelines to identify the top 10 core acoustic features. These features were subsequently examined within a new multiverse analysis framework to assess their robustness across analytical specifications.

**Results:**

Predictive performance was highly sensitive to analytical choices, with AUC values ranging from near chance (0.508) to high discriminative accuracy (0.856). Despite this variability, a core subset of acoustic features—including fundamental frequency (F0), F0 envelope, and Mel-frequency cepstral coefficients (MFCCs)—demonstrated robust and stable differences between the group screening positive for suicidal ideation and the screening-negative control group. These features remained statistically significant in 237 of 240 eligible specifications (98.8%).

**Conclusion:**

Although speech-based computational prediction of group status defined by suicidal ideation screening measures is highly dependent on analytical decisions, the discriminative acoustic features derived from machine learning remain remarkably stable, while it is important to recognize that observed acoustic differences likely reflect a combination of suicidal ideation, depression, anxiety, and general distress rather than a single underlying construct.

## 1 Introduction

Suicide is a global public health crisis, it is a tragedy that affects families, communities, and entire countries, leaving lasting effects on the relatives and friends of the deceased (World Health Organization, 2025). It's one of the most significant public health problems globally, contributing hundreds of thousands of deaths annually and imposing a substantial individual and societal burden (Seyedsalehi et al., 2025). Among college students, suicide represents a particularly pressing concern, for it is a major cause of unnatural death among college students. According to a China mental health survey, approximately 280,000 people commit suicide each year, among whom 40% suffer from depression, and 50% of the depressed patients are college students (Lu et al., 2021). The suicide rate among college students is two to four times higher than that of other age groups (Li et al., 2014). Currently, clinical practice primarily relies on self-report questionnaires and structured interviews for risk assessment (Baek et al., 2021). While these approaches have demonstrated some utility, they are subject to notable limitations: their effectiveness is influenced by participants' insight, social desirability, and recall biases, and assessments are typically conducted at discrete time points, making continuous, dynamic monitoring challenging (Hom et al., 2016; Deming et al., 2021; Haghish et al., 2024). These limitations have accelerated the search for objective, non-invasive correlates that can be collected continuously, positioning computational mental health as a vital frontier for innovation.

Speech, as a rich channel of emotional and cognitive expression, offers a promising avenue for objective risk assessment. Acoustic features—such as fundamental frequency (F0), jitter, shimmer, Mel-frequency cepstral coefficients (MFCCs)—capture subtle variations in vocal production that reflect underlying psychological states (Albuquerque et al., 2021; Silva et al., 2024). Recent advances in machine learning have enabled increasingly accurate prediction of suicide ideation from speech. For example, studies have reported classification accuracies ranging from 75 to 86% using acoustic and linguistic features in varied populations, including crisis hotline callers and military veterans (Belouali et al., 2021; Su et al., 2025). Despite these encouraging results, the field remains constrained by a reliance on single, optimized analytical pipelines. Most findings are derived from one specific combination of preprocessing, feature selection, and algorithm, which obscures the robustness and generalizability of the identified acoustic correlates (Ehtemam et al., 2024; Marie et al., 2026). This “single universe” approach elevates the risk of conclusion bias, limits reproducibility, and ultimately delays the translation of research into reliable clinical tools (Ciobanu-Caraus et al., 2024; D'Amour et al., 2022).

To address these methodological uncertainties, the multiverse analysis framework has emerged as a rigorous alternative for evaluating the stability of scientific findings across plausible analytical choices (Steegen et al., 2016). Rather than seeking a single best pipeline, multiverse analysis systematically explores a wide range of reasonable analytical decisions, thereby mapping how conclusions depend on specific preprocessing, modeling, and inference choices (Steegen et al., 2016; Carbine and Clayson, 2025). This approach has been successfully applied across diverse fields to enhance transparency and robustness. In psychology, multiverse analysis has been used to examine the relationship between depression and inflammatory correlates, revealing how analytical flexibility can substantially affect effect sizes and significance (Rengasamy et al., 2023). In neuroimaging, guided multiverse studies have exposed how variations in preprocessing and statistical modeling influence brain behavior correlations (Dafflon et al., 2022). Similarly, in clinical research, multiverse techniques have been employed to assess the robustness of treatment effects and to identify sources of bias in outcome measurement (Moore and Demeyere, 2022; Plessen et al., 2023). These applications demonstrate the method's capacity to differentiate robust signals from analytical artifacts and to improve the credibility of empirical claims.

In the context of speech-based suicide ideation prediction, however, the comprehensive application of multiverse analysis remains nascent. To date, no study has systematically evaluated how the interplay of feature processing, selection, and classifier choice impacts both model performance and the stability of acoustic feature. This gap constrains the identification of truly reliable vocal correlates and impedes the development of generalizable, clinically applicable tools.

To bridge this gap, the present study aims to systematically map and evaluate the robustness of acoustic feature–based predictions of group status defined by suicidal ideation screening. Rather than identifying a single optimal pipeline, we introduce a multiverse analysis framework that systematically examines 1,764 analytical specifications, encompassing preprocessing strategies, feature selection methods, and classification algorithms. Using this framework, we test two key hypotheses regarding robustness:

*Hypothesis 1 (model robustness): When models are trained on balanced samples, the predictive performance will be consistently high across the majority of analytical specifications, with limited variability*.

*Hypothesis 2 (feature robustness): The acoustic features that emerge as most important will show consistent statistical significance and effect direction across a wide range of analytical choices*.

By shifting the focus from optimizing a single pipeline to evaluating the robustness of both predictive performance and feature importance, this study aims to validate the effectiveness and stability of acoustic features in predicting suicide ideation. This approach enhances methodological transparency and facilitates the identification of stable and interpretable risk correlates.

## 2 Method

### 2.1 Speech materials

Sixteen reading prompts were developed and validated in a separate, independent stage to ensure suitability for Chinese university students. A distinct cohort of 54 university students (aged 18–25 years) rated an initial pool of 50 candidate sentences. These 54 participants were mutually exclusive from the main study cohort. These sentences were constructed to span three affective domains: positive-valence (imbued with Chinese traditional virtues and socialist core values to enhance cultural resonance), negative-valence expressions, and neutral factual statements. Syntactic moods (declarative, interrogative, exclamatory) were varied to elicit diverse prosodic patterns, and sentence length was capped at ≤ 100 characters with an estimated reading duration under 30 s to minimize fatigue.

Participants rated each sentence on nine-point Likert scales for pleasure, arousal, and dominance. Two licensed clinical psychologists then independently evaluated the sentences for readability, emotional expressiveness, and categorical balance. Based on consensus, 12 sentences (four per valence domain) were selected. Four additional suicide-related statements, adapted from validated instruments, were added to enhance clinical relevance, yielding a final set of 16 standardized prompts (Supplementary Table S1). These 16 prompts were subsequently used in the main study for the read speech task.

### 2.2 Participants and screening procedures

**Figure 1:**
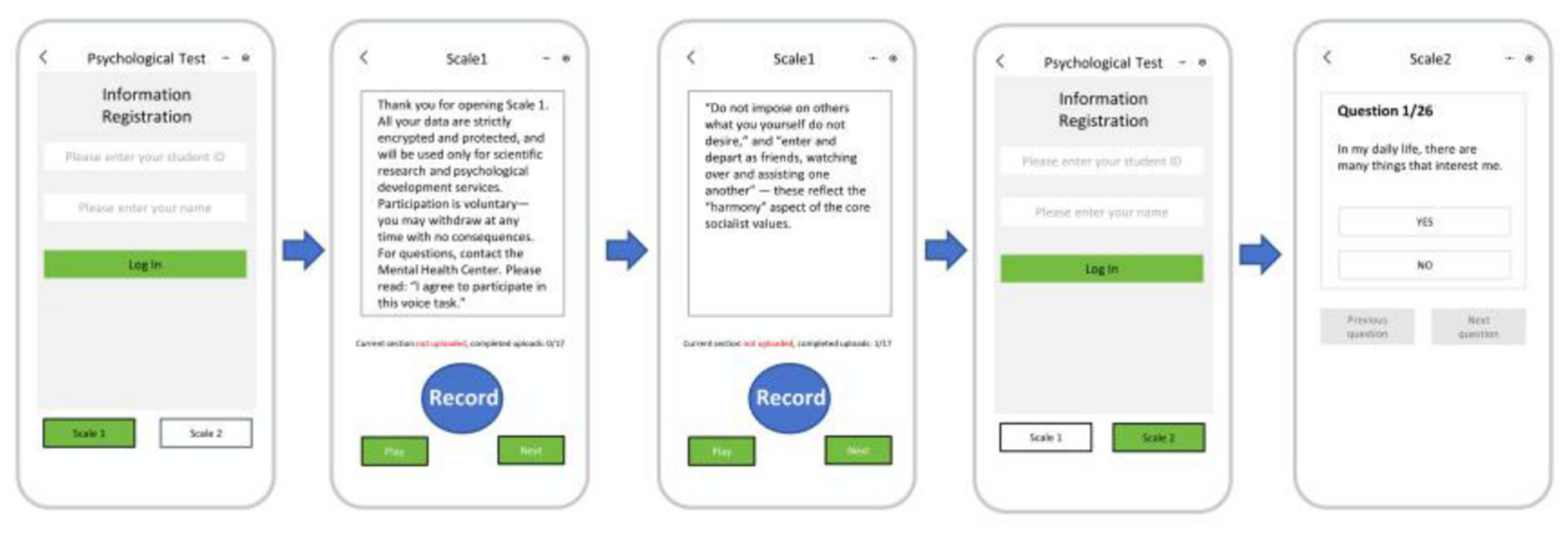
Screenshot of the WeChat mini-program interface.

This study employed a case–control design with a two-phase screening-and-confirmation procedure. Data were collected through a WeChat mini-program, yielding an initial pool of 747 questionnaire responses and 771 participants who submitted speech recordings. A screenshot of the program interface is shown in Figure 1; the original Chinese text was translated into English for the purpose of this figure. Participants completed speech recordings on their personal smartphones in familiar, low-stress settings to enhance ecological validity and comfort. All audio files were stored in encrypted, access-controlled repositories and used exclusively for acoustic feature extraction.

After excluding incomplete submissions and conducting audio quality screening, 715 participants remained eligible for suicidal ideation (SI) assessment. SI was assessed using the Self-Rating Idea of Suicide Scale (SIOSS), a 26-item self-report scale developed for the Chinese population that comprises four factors (despair, sleep, optimism, and concealment) and has demonstrated adequate reliability and validity (Xia et al., 2012, 2007). A threshold of ≥12 was used to indicate positive screens. To minimize misclassification due to response concealment, individuals scoring ≥4 on the SIOSS concealment subscale were excluded. Participants who screened positive subsequently underwent a face-to-face follow-up assessment conducted by trained counselors in a university counseling center. All counselors held national-level qualifications in psychological counseling. The assessment employed the Chinese version of the Columbia–Suicide Severity Rating Scale (C-SSRS) Screening Version (Bjureberg et al., 2022; Xiao et al., 2025). Participants who met the follow-up criteria were referred to hospitals for further evaluation and intervention. This process identified 48 confirmed SI cases.

From the pool of participants who screened negative on the SIOSS (< 12), a control group of 48 individuals was selected using 1:1 random sampling. Age and sex were compared between groups and were treated as covariates in the primary analysis. The final analytic sample comprised 96 participants (aged 16–25 years; 55 males), each of whom completed a standardized read speech task in a naturalistic environment, producing 16 recordings per participant. This resulted in a total of 1,536 audio files for subsequent feature extraction and model development.

The study was reviewed and approved by the Ethics Committee of the Fourth People's Hospital of Chongqing [Approval No. (2025) Ethical Review No. (73)]. All participants provided written informed consent in accordance with the Declaration of Helsinki.

### 2.3 Preprocessing of audio recordings

We implemented a standardized preprocessing pipeline. Raw recordings were first denoised using the non-destructive Model Scope Acoustic Noise Suppression (ANS) model optimized with multiple loss functions, and subsequently transcribed with the Google Speech-to-Text Application Programming Interface (API) with automatic language detection. Transcripts were aligned with predefined target texts using a custom dual-weighted fuzzy-matching algorithm (FuzzyWuzzy; 60% token set similarity and 40% edit-distance similarity), with a composite score >65% indicating valid matches; all matches were manually reviewed, and ambiguous cases were corrected by trained researchers. Finally, recordings were converted from MP3 (44.1 kHz stereo, 128 kbps) to Waveform Audio File Format (WAV) (16 kHz mono, 16-bit), using ffmpeg. At the constant bitrate of 128 kbps, MP3 compression negligibly degrades the acoustic features used in this study, whereas the non-destructive denoising step improves signal-to-noise ratio and may enhance the robustness of prosodic and energy-related features (Cavalcanti et al., 2023; Oreskovic et al., 2024).

### 2.4 Feature extraction

Acoustic features were extracted using openSMILE (version 3.0.2; audEERING GmbH, Gilching, Germany) by combining the emobase and emobase2010 feature sets (Eyben et al., 2010). This yielded 570 features derived from 30 low–level descriptors (LLDs), each summarized by 19 statistical functionals; the complete list of features is provided in Supplementary Data S1. The selected acoustic features capture distinct aspects of vocal production that have established links to psychological states: fundamental frequency (F0) and its variability reflect emotional arousal and stress response; jitter and shimmer index vocal fold control and are sensitive to anxiety and physiological tension; Mel–frequency cepstral coefficients (MFCCs) encode timbral and spectral properties associated with cognitive load and affective valence; and power spectral density (PSD) captures energy distribution across frequency bands, which may shift under depressive or fatigued states (Albuquerque et al., 2021; Kappen et al., 2022; Silva et al., 2024). These features collectively provide a multivariate representation of speech that is theoretically relevant to suicidality related alterations in emotion and cognition.

### 2.5 Multiverse analysis design rationale

To systematically evaluate the robustness of conclusions drawn from acoustic based prediction of suicidal ideation, we implemented a multiverse analysis framework that varies four key dimensions of the machine learning pipeline: (1) preprocessing methods (standardization vs. normalization), which influence feature scaling and model convergence; (2) feature selection methods (filter, wrapper, embedded, and fusion strategies), which determine the subset of features used for modeling; (3) number of selected features (ranging from 10 to 30), controlling for dimensionality and overfitting; and (4) classification models (seven widely used algorithms), representing diverse inductive biases and functional forms. These dimensions were chosen because they encompass the major decision points in a typical speech–ML workflow—from data preparation and feature refinement to model building—each of which can substantially affect performance and interpretability. By exhaustively combining these choices, our design allows a panoramic assessment of how analytical variability propagates through the pipeline and which findings remain stable across plausible specifications.

### 2.6 Hypothesis operationalization

Hypothesis 1a (model effectiveness): When models are trained on balanced samples, the mean test-set area under the receiver operating characteristic curve (AUC) across the 1,764 analytical specifications will be ≥0.70.

The mean AUC was calculated as:

$$\begin{array}{l} Mean\;AUC=\frac{1}{N}\overset{N}{\underset{i=1}{\sum }}AUC_{i}\;\geq \;0.70 \end{array}$$

*where N* = *1,764 denotes the total number of analytical specifications*, *AUC*_*i*_ *is the AUC value obtained for the i-th specification. A mean AUC* ≥*0.70 was considered to indicate model effectiveness*.

Hypothesis 1b (model stability): When models are trained on balanced samples, the standard deviation of the 1,764 test-set AUCs around their mean will not exceed 0.0485.

The standard deviation was calculated as:

$$\begin{array}{l} SD=\sqrt{\frac{1}{N}\overset{N}{\underset{i=1}{\sum }}{\left(AUC_{i}-\bar{AUC}\right)}^{2}\;\;\;\;\;\;}\;\leq 0.0485 \end{array}$$

*Where N* = *1,764 denotes the total number of analytical specifications*, *AUC*_*i*_ *is the AUC value obtained for the i-th specification, and* $\bar{AUC}$ *represents the mean AUC across all specifications. A standard deviation* ≤ *0.0485 was considered indicative of robust model performance*.

Hypothesis 2a (feature effectiveness): The 10 acoustic features with the highest average importance collectively form a feature-level multiverse framework. Within the 240 analytical specifications of this framework, at least 80% are expected to show statistical significance (*p* ≤ 0.05).

The proportion of significant specifications was calculated as:

$$\begin{array}{l} Proportion\;of\;significant\;specifications=\frac{\overset{N}{\underset{i=1}{\sum }}1_{\left\{p_{i}\leq 0.05\right\}}}{N}\;\geq 0.80 \end{array}$$

*Where N is the number of specifications*, 1_{_*p*__*i*_ ≤ 0.05}_ *is an indicator function that equals 1 if the i-th specification is statistically significant, and 0 otherwise. A proportion of significant specifications*≥*0.80 was considered supportive of the hypothesis*.

Hypothesis 2b (feature stability): The 10 acoustic features with the highest average importance collectively form a feature-level multiverse framework. For each feature, at least 95% of the analytical specifications are expected to show consistent effect directions, as indicated by the sign of standardized β coefficients.

Feature stability for a given feature f was calculated as:

$$\begin{array}{l} Consistency\;\;f=\frac{\max\left(\overset{N}{\underset{i=1}{\sum }}1_{\left\{\beta_{f,j}\geq 0\right\}},\overset{N}{\underset{i=1}{\sum }}1_{\left\{\beta_{f,j}<0\right\}}\right)}{N}\;\geq 0.95 \end{array}$$

*Where N* = *240 denotes the total number of analytical specifications*, β_*f, j*_ *is the standardized coefficient for feature f in the i-th specification, and* 1_{•}_ *is an indicator function that equals 1 if the condition holds and 0 otherwise. A consistency value* ≥*0.95 was considered to reflect feature stability*.

### 2.7 Machine learning multiverse analysis

A full factorial combination of the above dimensions yielded 1,764 analytically plausible modeling paths. We adopted a repeated subject-level stratified five-fold cross-validation scheme with five repeats. Within each repeat, the same fold assignments were used across all modeling paths to ensure fair comparability. All recordings from the same participant were kept within a single fold to guarantee independence between training and test sets. Participants were randomly assigned to folds while maintaining balance in suicide ideation status and sex. All preprocessing, feature selection, and model training were performed strictly within each training fold, with parameters then applied to the held-out test data to prevent leakage.

### 2.8 Data preprocessing

Constant features were removed based on training data. To reduce multicollinearity, one feature from each highly correlated pair (|*r*| > 0.90) was excluded. Remaining features were scaled via either *Z*–score standardization (using training mean and SD) or min–max normalization (using training min and max), with the same transformation applied to test data.

### 2.9 Feature selection

Six selection methods were implemented: two filter-based [Correlation, analysis of variance (ANOVA)], one wrapper–based (Random Forest importance, RF), two embedded [least absolute shrinkage and selection operator (LASSO), Elasticnet], and a custom Fusion strategy that aggregated rankings from RF, Correlation, and ANOVA. All selection was performed on training folds only, and the number of retained features *k* varied from 10 to 30.

### 2.10 Model training and evaluation

For each feature subset, seven classifiers were trained: Support Vector Machine (SVM), Random Forest (RF), XGBoost (XGB), logistic regression, K-Nearest Neighbors (KNN), Naive Bayes (NB), and Neural Networks (NNET). Generalization performance was evaluated on the independent test fold using AUC, accuracy, sensitivity, specificity, precision, and F1–score. All analyses were conducted in R using standard packages, including “randomForest (R package; Andy Liaw and Matthew Wiener, available through CRAN, Vienna, Austria),” “glmnet (R package; Jerome Friedman, Trevor Hastie, and Robert Tibshirani, available through CRAN, Vienna, Austria),” “xgboost (R package; Tianqi Chen et al., available through CRAN, Vienna, Austria),” “e1071,” “caret (R package; Max Kuhn, available through CRAN, Vienna, Austria),” and “naivebayes (R package; Michal Majka, available through CRAN, Vienna, Austria).”

Given the relatively small sample size (*N* = 96), we adopted a conservative, generalization-oriented training strategy. Hyperparameter settings were largely kept at package defaults or fixed to commonly recommended values, with only limited adjustments to ensure numerical stability. Below we report only the non-default settings that were explicitly adjusted:

*RF: number of trees set to 300, minimum node size set to 10, all other parameters at package defaults*.

*Logistic regression: elastic-net mixing parameter* α = *0.5 (L1/L2 balance); regularization strength* λ *selected via five-fold cross-validation within the training data*.

*XGB: max tree depth* = *3, learning rate (eta)* = *0.1, subsample* = *0.8, L1 regularization (alpha)* = *0.5, L2 regularization (lambda)* = *1, all other parameters at package defaults*.

All models were trained on training folds only, and performance was evaluated on fully independent test folds. Any parameter selection procedures were strictly nested within the training data to prevent information leakage. Our design ensures that variability in model performance reflects robustness to analytical choices rather than optimization-driven inflation.

### 2.11 Feature level multiverse analysis

Based on the machine learning multiverse results, we identified the top 10 most robust features by integrating their importance scores, contributions to AUC, and selection frequency across specifications. For each feature f, we calculated a comprehensive importance score as:

$$\begin{array}{lll} {Comprehensive\;Importance\;Score}_{f} & = & \frac{1}{3}\times {\bar{Imp}}_{f}+\frac{1}{3}\times {\bar{AUC}}_{f}\\ +\frac{1}{3}\times {\bar{Freq}}_{f} \end{array}$$

*Where* ${\bar{Imp}}_{f}$ *is the mean normalized importance score of feature f across all specifications (with importance normalized to [0,1] within each specification)*, ${\bar{AUC}}_{f}$ *is the mean model AUC when feature f was selected, and* ${\bar{Freq}}_{f}$ *is the proportion of specifications in which feature f was selected*.

We then constructed a second stage multiverse to examine the stability of these features' associations with suicidal ideation. This feature multiverse varied four dimensions: (1) the 10 selected features, (2) feature processing (raw, standardized, normalized), (3) covariate adjustment (none, sex, age, sex+age), and (4) outcome modeling (binary logistic regression vs. continuous linear regression). This yielded 240 analytical specifications, enabling a systematic evaluation of how inferential conclusions depend on statistical choices.

## 3 Result

### 3.1 Participant characteristics

A total of 96 participants (48 per group) were included in the final sample, ranging in age from 16 to 25 years (M = 18.76, SD = 1.48). The overall proportion of males was 57.3% (55/96). As shown in Table 1, no significant group difference was observed in gender distribution (χ^2^ = 0.17, *p* = 0.68). However, the groups differed significantly in age, with the SI group showing a higher mean age (19.25 ± 1.78 years) compared to the control group (18.27 ± 0.92 years; *t*_(94)_ = −3.39, *p* = 0.001).

**Table 1:** Demographics of participants.

Variable	Overall (*N* = 96)	SI group (*n* = 48)	Control group (*n* = 48)	*p-*Value
Age, years (M ± SD)	18.76 ± 1.48	19.25 ± 1.78	18.27 ± 0.92	0.001
Sex, *n* (%)				
Male	55 (57.3)	29 (60.4)	26 (54.2)	0.68
Female	41 (42.7)	19 (39.6)	22 (45.8)	

### 3.2 Machine learning multiverse analysis

**Figure 2:**
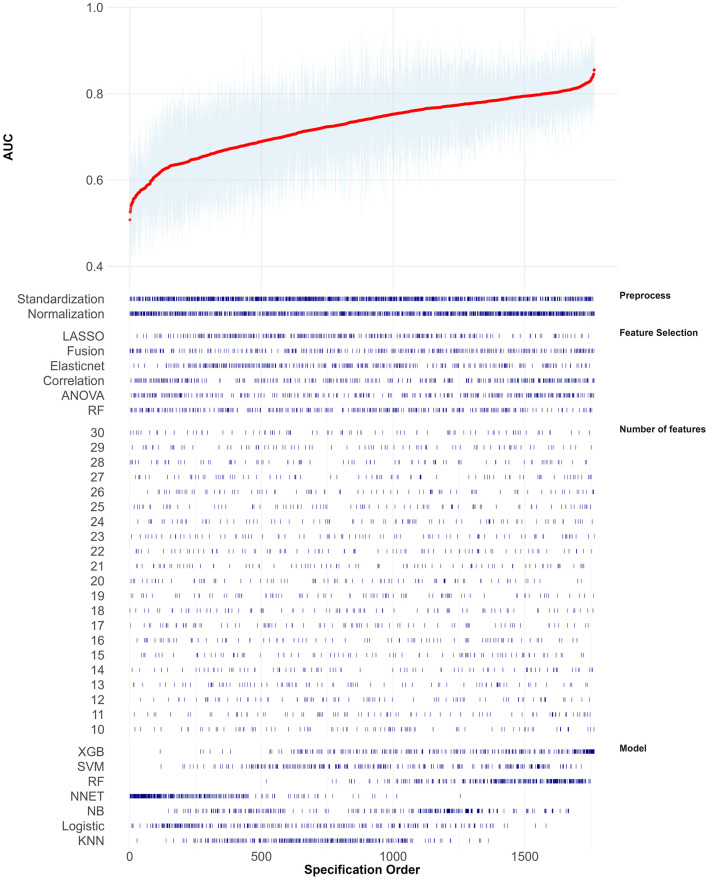
Specification curve and raincloud plot of the machine learning multiverse analysis. The figure consists of two panels. The upper panel shows the specification curve, where each data point represents the area under the curve (AUC) for a unique analytic specification. Specifications are ordered on the *x*-axis by increasing AUC, with the corresponding AUC values plotted on the *y*-axis. The lower panel is a raincloud plot that visualizes the analytic decisions underlying each specification. The *x*-axis corresponds to the specification number (aligned with the order in the upper panel). On the right, the four decision dimensions are listed: feature preprocessing approach, feature selection tactic, number of selected features, and classifier category. On the left, the specific choice made for each dimension is shown. Each specification in the upper panel is linked to its four corresponding decision points in the lower panel.

To conduct a systematic assessment of the influence of analytical selections on predictive performance, specification curve plots were constructed for the Area Under the Curve (AUC), which was employed as the primary performance indicator (Figure 2). Specification curves for the remaining metrics, namely accuracy, sensitivity, specificity, precision, and F1-score, are presented in Supplementary Figures S1–S5. Each specification curve showcases the ranked performance of all 1,764 model specifications for the corresponding metric, and the associated combinations of analytical selections are depicted along the bottom of the plot. Descriptive statistics for all six metrics, encompassing the mean, minimum, median, and maximum values, are summarized in Table 2.

**Table 2:** Model performance metrics in all specifications.

Metric	Mean	Min	Median	Max
AUC	0.727	0.508	0.739	0.856
Accuracy	0.666	0.329	0.681	0.788
F1-score	0.657	0.312	0.668	0.789
Sensitivity	0.677	0.295	0.691	0.891
Specificity	0.672	0.345	0.685	0.87
Precision	0.664	0.295	0.675	0.84

Across all 1,764 analytically tenable specifications, the mean and median values of model performance levels were comparatively high (mean AUC = 0.727; SD = 0.065). This result supports the hypothesis 1a (model effectiveness), as the mean AUC exceeded the specified threshold of 0.7. However, the hypothesis 2b (model stability) was not supported, as the standard deviation (SD = 0.065) surpassed the predefined criterion of ≤ 0.0485, indicating greater variability in model performance across specifications. These results indicate that the model was effective, supporting Hypothesis 1a, yet lacked stability, failing to support Hypothesis 1b. Thus, the hypothesized model robustness was not achieved, suggesting that diverse analytic selections had a notable impact on predictive performance.

To further clarify the sources of performance variability, we investigated the sensitivity of various machine learning models to analytic choices. As shown in Table 3, different choices within the same dimension exhibit significant differences in predictive performance and robustness, with the most pronounced differences observed for the model dimension.

**Table 3:** Summary statistics of AUC values across analytic specifications.

Dimension	Choice	Count	Mean AUC	Min AUC	Median AUC	Max AUC
Preprocess	Standardization	882	0.723	0.526	0.729	0.840
	Normalization	882	0.731	0.508	0.749	0.856
Feature selection	LASSO Fusion	294 294	0.721 0.732	0.566 0.508	0.723 0.753	0.827 0.855
	Elasticnet	294	0.724	0.557	0.727	0.829
	Correlation	294	0.733	0.539	0.757	0.856
	ANOVA	294	0.731	0.542	0.757	0.846
	RF	294	0.719	0.529	0.737	0.837
Number of features	30 29	84 84	0.720 0.724	0.534 0.547	0.729 0.742	0.828 0.835
	28	84	0.720	0.526	0.738	0.825
	27	84	0.727	0.560	0.746	0.832
	26	84	0.728	0.588	0.732	0.846
	25	84	0.727	0.557	0.739	0.830
	24	84	0.730	0.568	0.751	0.838
	23	84	0.727	0.544	0.738	0.856
	22	84	0.725	0.564	0.735	0.836
	21	84	0.725	0.565	0.736	0.821
	20	84	0.719	0.539	0.737	0.818
	19	84	0.725	0.542	0.743	0.845
	18	84	0.723	0.508	0.733	0.840
	17	84	0.717	0.529	0.729	0.826
	16	84	0.722	0.566	0.742	0.813
	15	84	0.733	0.577	0.752	0.821
	14	84	0.734	0.546	0.733	0.838
	13	84	0.724	0.556	0.725	0.825
	12	84	0.732	0.575	0.736	0.829
	11	84	0.741	0.558	0.750	0.829
	10	84	0.734	0.559	0.748	0.855
Model	XGB	252	0.773	0.618	0.779	0.856
	SVM	252	0.744	0.619	0.746	0.818
	RF	252	0.789	0.692	0.792	0.830
	NNET	252	0.631	0.508	0.634	0.775
	NB	252	0.738	0.629	0.763	0.809
	Logistic	252	0.698	0.546	0.698	0.800
	KNN	252	0.713	0.566	0.717	0.783

Among all the models, the RF model exhibited superior stability and comprehensive performance. The RF model achieved the highest median value of the AUC, reaching 0.792, and presented the most concentrated performance distribution. Specifically, it had a minimum AUC of 0.692, a maximum AUC of 0.830, and the smallest standard deviation across specifications (*SD* = 0.021). This phenomenon indicates that the RF model maintained consistently high discriminative ability across a wide range of feature selection and preprocessing combinations.

In contrast, the XGB model demonstrated the greatest performance potential but was also the most sensitive to analytical selections. Although the XGB model attained the highest maximum AUC value of 0.856 among all models, its performance distribution was the broadest, with a minimum AUC of 0.618 and a range of 0.237. This implies that its optimal performance was highly dependent on meticulously adjusted analytical settings.

The SVM, NB, and KNN models exhibited a certain level of robustness, with median AUC values ranging from 0.717 to 0.746, and notable variability was observed across different specifications. In contrast, the logistic regression and NNET models were highly sensitive to analytical selections, with the minimum AUC values dropping to 0.546 and 0.508, respectively. This indicates that certain analytical methods yielded discriminative performance approaching the random chance level.

**Figure 3:**
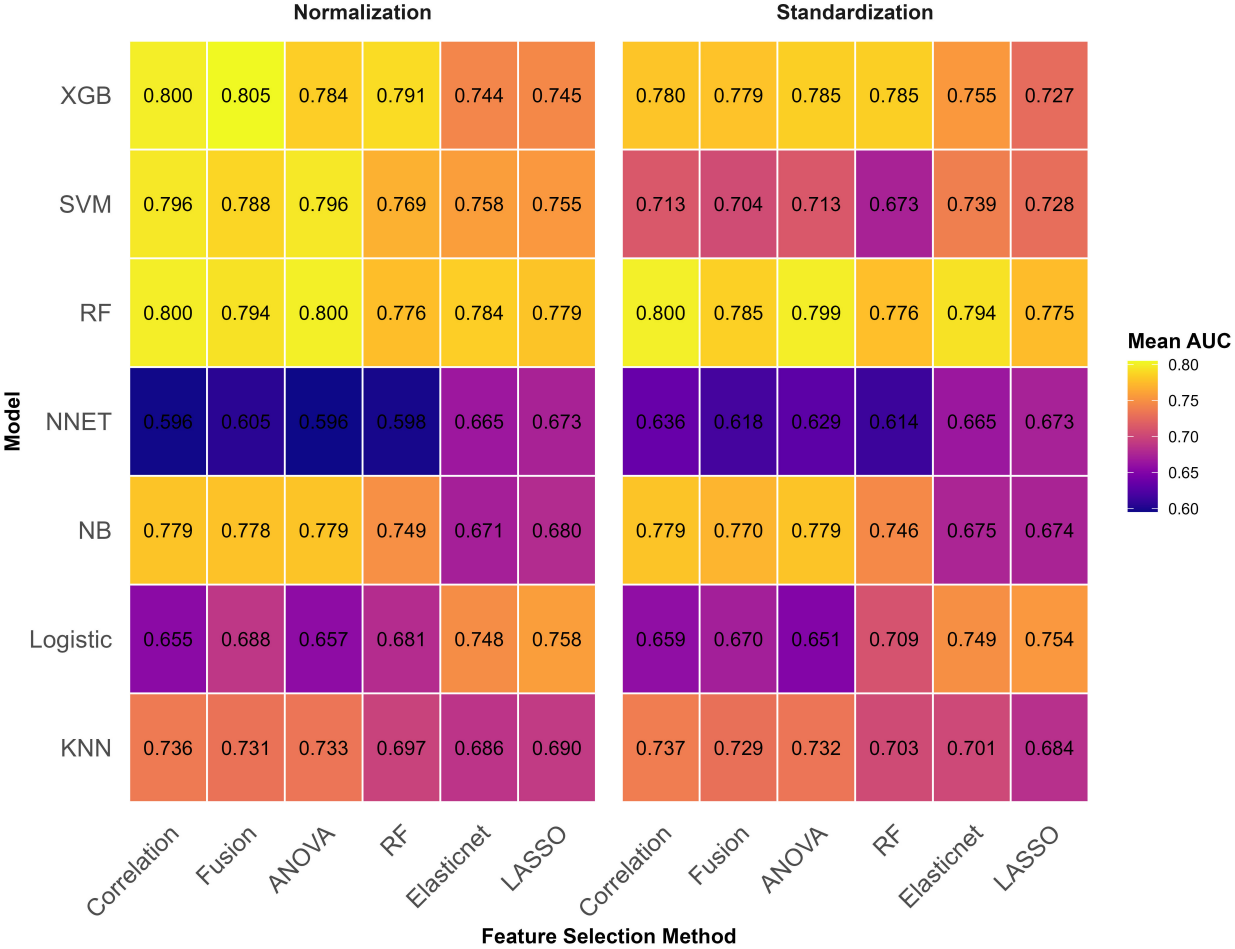
Heatmaps depicting the performance of models under diverse feature scaling strategies. The figure comprises two panels, corresponding to feature standardization and normalization, respectively. Each heatmap showcases the average predictive performance of combinations of machine learning models and feature selection methods under a specific feature scaling strategy. The *x* axis denotes the feature selection method, while the *y* axis represents the type of machine learning model. The intensity of color signifies the average value of the performance metric, aggregated over all remaining analytical decisions. This figure aims to assess the consistency of model performance patterns across different feature scaling options, thus evaluating the robustness of the results.

To decompose the sources of disparities in model robustness and to identify crucial analytic decision-making points, we employed a heatmap to visualize the associations between analytic choices and model performance (Figure 3). This visualization offers an intuitive representation of the intricate interaction effects among machine learning models, feature selection strategies, and data-scaling methods.

Firstly, the heatmap directly validates the observed robustness disparities across models. The row corresponding to the RF model demonstrates consistently darker and more uniform coloration throughout the entire matrix. This indicates that the performance of the RF model remains stably high, with an AUC ranging from 0.775 to 0.800 across nearly all analytic configurations. In stark contrast, the rows for the SVM and NNET models display significant color variability, which reflects a strong dependence of their performance on specific analytic choices.

Secondly, the analysis clarifies the specific sources of model sensitivity. For the SVM model, the selection of the data-scaling method emerges as the primary factor determining performance, as different scaling approaches result in considerable fluctuations in AUC. In contrast, tree-based models (RF and XGB) appear to be largely insensitive to feature scaling. Meanwhile, the choice of feature selection strategy has a more substantial impact on the final performance of the logistic regression and KNN models.

Within the framework of machine learning multiverse analysis, feature importance estimation methods were chosen according to the characteristics of each model. As shown in Table 4, by integrating the mean importance of each feature, its contribution to the AUC, and its selection frequency across all analytical specifications, a composite feature importance score was calculated. Based on this score, the top 10 most robust features were recognized and retained for subsequent core analyses.

**Table 4:** Feature rankings based on a comprehensive importance score.

Feature	Mean importance	Frequency	Mean AUC	Comprehensive importance
F0_sma_iqr2-3	0.191	0.526	0.724	0.480
F0env_sma_stddev	0.147	0.512	0.728	0.462
mfcc_sma[12]_kurtosis	0.117	0.403	0.714	0.411
F0env_sma_iqr1-2	0.046	0.367	0.714	0.376
mfcc_sma[5]_kurtosis	0.076	0.330	0.701	0.369
F0env_sma_linregerrQ	0.041	0.308	0.714	0.354
mfcc_sma[2]_min	0.039	0.258	0.730	0.342
F0_sma_stddev	0.051	0.205	0.751	0.336
F0env_sma_linregc1	0.048	0.201	0.737	0.329
mfcc_sma[3]_kurtosis	0.049	0.243	0.689	0.327
pcm_intensity_sma_iqr1-3	0.029	0.195	0.741	0.322
pcm_loudness_sma_maxPos	0.040	0.185	0.739	0.321
pcm_loudness_sma_skewness	0.032	0.186	0.741	0.320
pcm_loudness_sma_iqr1-3	0.033	0.190	0.733	0.319
pcm_loudness_sma_kurtosis	0.031	0.181	0.740	0.317
HNR_sma_kurtosis	0.061	0.177	0.707	0.315
HNR_sma_iqr1-2	0.048	0.194	0.702	0.315
mfcc_sma[1]_maxPos	0.030	0.154	0.739	0.307
lspFreq_sma[1]_stddev	0.044	0.000	0.868	0.304
mfcc_sma[1]_minPos	0.025	0.142	0.738	0.302
lspFreq_sma[5]_quartile2	0.035	0.143	0.727	0.302
lspFreq_sma[5]_quartile3	0.036	0.161	0.705	0.301
pcm_intensity_sma_maxPos	0.038	0.038	0.825	0.300
HNR_sma_stddev	0.048	0.137	0.715	0.300
lspFreq_sma[5]_skewness	0.046	0.074	0.780	0.300
mfcc_sma[9]_quartile3	0.030	0.000	0.868	0.300
F0env_sma_kurtosis	0.038	0.047	0.808	0.298
F0_sma_kurtosis	0.062	0.034	0.792	0.296
F0_sma_linregc1	0.060	0.036	0.789	0.295
mfcc_sma[9]_minPos	0.103	0.097	0.682	0.294

### 3.3 Feature multiverse analysis

**Figure 4:**
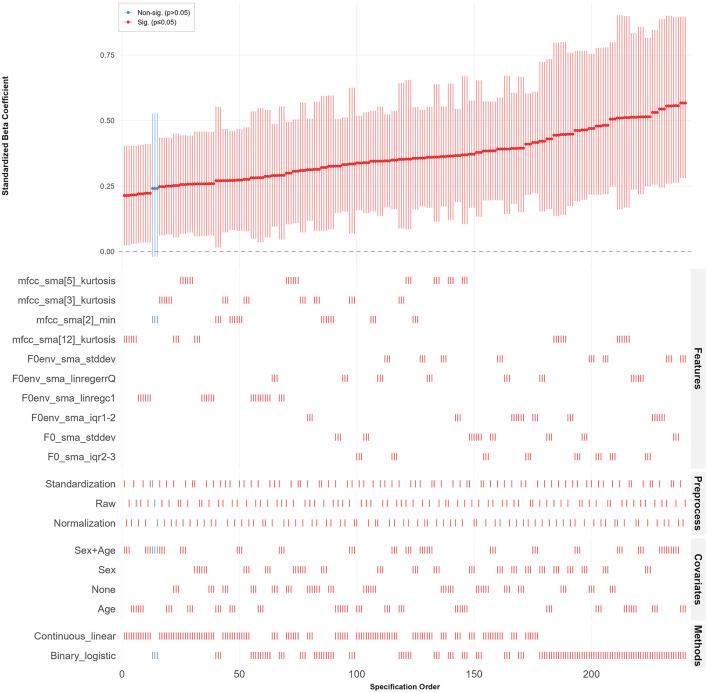
Specification curve and raincloud plot for features in the multiverse analysis (standardized β). The figure consists of two panels. The upper panel shows the specification curve, where each data point represents the standardized coefficient (β) for a given analytic specification. Specifications are ordered on the *x*-axis by increasing β, with the corresponding β values plotted on the *y*-axis. The lower panel is a raincloud plot that visualizes the analytic decisions underlying each specification. The *x*-axis corresponds to the specification number (aligned with the order in the upper panel). On the right, the four decision dimensions are listed: feature, preprocess, covariates, methods. On the left, the specific choice made for each dimension is shown. Each specification in the upper panel is linked to its four corresponding decision points in the lower panel.

Multiverse analyses at the feature level revealed that, when focusing on a given feature and holding covariates and model structure constant, varying the feature value processing method (raw, standardization, or normalization) did not change either the standardized effect size or the associated *p*-value. This phenomenon is visually presented in the specification curve (Figure 4) and further quantified in Table 5, which summarizes the distribution of effect estimates across all analytically feasible specifications, grouped by key analytical dimensions. As shown in the table, the mean standardized effect sizes for raw, normalization, and standardization were identical (0.36), and the distributions across other dimensions remained stable. These results indicate that feature robustness is an inherent characteristic of the feature itself, and that the strength of the association between the feature and the outcome variable is independent of scale transformation.

**Table 5:** Summary of standardized effect sizes (β) across analytical specifications.

Dimension	Choice	Count	Mean β	Min β	Median β	Max β
Feature	mfcc_sma[5]_kurtosis	24	0.32	0.26	0.33	0.37
	mfcc_sma[3]_kurtosis	24	0.29	0.25	0.29	0.35
	mfcc_sma[2]_min	24	0.3	0.24	0.3	0.36
	mfcc_sma[12]_kurtosis	24	0.36	0.21	0.35	0.51
	F0env_sma_stddev	24	0.44	0.35	0.43	0.57
	F0env_sma_linregerrQ	24	0.4	0.29	0.38	0.51
	F0env_sma_linregc1	24	0.26	0.22	0.27	0.29
	F0env_sma_iqr1-2	24	0.43	0.31	0.41	0.54
	F0_sma_stddev	24	0.41	0.33	0.38	0.56
	F0_sma_iqr2-3	24	0.43	0.34	0.44	0.51
Process	Standardization	80	0.36	0.21	0.35	0.57
	Raw	80	0.36	0.21	0.35	0.57
	Normalization	80	0.36	0.21	0.35	0.57
Covariates	Sex+Age	60	0.37	0.21	0.36	0.56
	Sex	60	0.37	0.26	0.37	0.51
	None	60	0.35	0.25	0.34	0.51
	Age	60	0.36	0.22	0.34	0.57
Methods	Continuous_linear	120	0.31	0.21	0.32	0.42
	Binary_logistic	120	0.41	0.24	0.43	0.57

Overall, the associations between the 10 features and the outcome variable demonstrated a high level of robustness. Statistical significance (*p* ≤ *0.0*5) was maintained in 98.8% (237/240) of all analytically feasible specifications, and the direction of effects (sign of β) remained fully consistent across all specifications. These results satisfy both hypothesis 2a (feature robustness) and hypothesis 2b (feature direction consistency), indicating that the selected features are both robust and directionally consistent.

As depicted in the specification curve, the effect sizes varied within a relatively narrow range across specifications, indicating relatively limited variability. For instance, regarding the core feature F0_sma_iqr2–3, the median β was 0.41, with the 95%CI [0.35, 0.52]. In stark contrast, the AUC values in the first-stage machine learning models ranged from near chance to excellent performance across specifications. This disparity offers compelling evidence that the acoustic features themselves convey robust discriminative signals, while the uncertainty in study conclusions predominantly stems from differences in downstream modeling and analytic selections.

Extending this analysis to sex-stratified subgroups, each with 120 analytical specifications, sharply reduced the sample size from 96 to 55 males and 41 females, which lowered statistical power and led to a decreased proportion of statistically significant results. Nonetheless, the direction of effects remained fully consistent across all 240 specifications, with a median β of 0.46 for males and 0.22 for females, further reinforcing the robustness of the feature-level associations, as detailed in the Supplementary Table S2.

## 4 Discussion

We implemented a multiverse analysis framework to systematically evaluate how analytical choices shape conclusions in acoustic-based detection of group differences defined by suicidal ideation screening. Our findings reveal a critical divergence: while predictive performance varied substantially across specifications, a core set of acoustic features selected via machine learning demonstrated remarkably stable associations with group status. Specifically, the mean test-set AUC across the 1,764 analytical specifications was 0.727, meeting the predefined criterion for model effectiveness (mean AUC ≥ 0.70). However, the standard deviation of 0.065 exceeded the predefined stability criterion ( ≤ 0.0485), indicating that the jointly defined standard for model robustness was not achieved. By contrast, the selected acoustic features remained statistically significant in 98.8% of analytical scenarios and showed fully consistent effect directions, thereby satisfying both feature-level hypotheses and demonstrating feature robustness. This divergence suggests that variability in model-level outcomes stems largely from pipeline-dependent decisions rather than instability in the underlying vocal signal, whereas the acoustic features themselves represent a more stable signal.

When interpreting these results, it is important to acknowledge that we cannot rule out the potential influence of unmeasured confounding factors—such as depression or general psychological distress, which have been shown to affect acoustic features in previous studies (Ozdas et al., 2004; Suwannakhun and Yingthawornsuk, 2019; Ruckchopsanti et al., 2025). Consequently, our study design does not allow us to attribute the acoustic differences specifically to suicidal ideation as a distinct construct, nor to draw precise boundaries between suicidal ideation and other emotional or mental states. Instead, the stable acoustic features identified here are more appropriately interpreted as reflecting differences between groups defined by screening measures—a grouping that likely encompasses a combination of suicidal ideation, depression, anxiety, and general distress. Below, we interpret these results in light of psychological mechanisms and methodological implications, outline limitations, and suggest future directions.

### 4.1 Interpretation of robust acoustic features

The high robustness of certain acoustic features—such as F0 and MFCC—suggests they may capture psychophysiological alterations integral to psychological states associated with suicide risk. For instance, increased F0 instability and elevated jitter are often linked to emotional dysregulation and stress induced autonomic arousal, reflecting sympathetic nervous system activation that modulates vocal fold tension (Kappen et al., 2022; Silva et al., 2024). This is further supported by studies linking F0 and vocal instability to suicidal risk (Venek et al., 2017; Figueroa Saavedra et al., 2021). Similarly, MFCC patterns, which encode timbral and resonant properties of speech, may correlate with reduced cognitive flexibility and psychomotor retardation observed in depressive states (Cummins et al., 2015; Lin et al., 2025; Wei et al., 2025). These features potentially index downstream effects of neurobiological changes, such as altered respiratory control, laryngeal muscle tone, and prosodic planning, that accompany suicidal thinking (Teixeira et al., 2008; Van Puyvelde et al., 2018). Their stability across analytical pipelines reinforces their validity as candidate correlates, less susceptible to analytical noise than performance metrics tied to a specific model configuration.

### 4.2 Sources of model performance variability

Beyond analytical choices, several data-inherent factors may have contributed to performance fluctuations. First, although we balanced groups by sex and screening status, the modest sample size (*n* = 96) and potential unmeasured heterogeneity within the suicidal ideation group (e.g., varying severity, chronicity, comorbid anxiety) could limit model generalizability and increase variance. Second, the use of read aloud speech may introduce less emotional variability compared to spontaneous speech, possibly attenuating discriminative power (Alghowinem et al., 2013; Chien et al., 2025). Third, despite rigorous cross-validation, variability in recording conditions—including device heterogeneity, environmental background, and participant behavior—was present in this naturalistic dataset; we attempted to mitigate its impact through consistent preprocessing pipelines and stratified cross-validation, but residual acoustic noise may still interact with preprocessing choices (Leem et al., 2024). To mitigate these issues in future studies, we recommend: (1) applying synthetic data augmentation or weighted learning to enhance robustness to imbalance; (2) integrating noise robust feature representations; (3) explicitly reporting environmental and participant-level covariates that could influence vocal acoustics; and (4) collect data in a controlled environment whenever possible.

### 4.3 Methodological implications

Toward Transparent Computational Psychiatry, our application of multiverse analysis highlights its utility for enhancing methodological rigor in psycholinguistic and clinical machine learning. By exhaustively varying preprocessing, feature selection, and modeling decisions, we shift the focus from identifying a single “best” pipeline to evaluating the stability of findings across plausible analytical landscapes (Steegen et al., 2016; Weermeijer et al., 2022). This approach is particularly valuable in fields such as computational psychiatry and speech correlate research, where heterogeneity in data processing can obscure true effects and impede replication (Mahapatra and Mahapatra, 2026). Moving forward, we encourage researchers to adopt multiverse as a standard robustness check, promoting transparency, facilitating cross study comparisons, and accelerating the translation of research into clinically reliable tools.

### 4.4 Limitations and future directions

Several limitations should be acknowledged. First, a key limitation is the absence of independent measures of depression, anxiety, and general psychological distress. Because the groups were defined solely by SIOSS and C-SSRS screening, the observed acoustic differences cannot be uniquely attributed to suicidal ideation or suicide risk. Instead, they reflect a composite group status that likely encompasses multiple overlapping psychological states. Second, our sample was drawn from a single university in China, restricting age, cultural, and linguistic diversity. Third, we relied exclusively on read speech tasks; spontaneous speech, which may better reflect natural affective and cognitive processes, should be included in future data collection. Fourth, our study did not account for several factors that may influence acoustic characteristics. These include medication use (particularly psychotropic medications), comorbid psychiatric conditions, substance abuse, and transient respiratory conditions such as cold or rhinitis. Additionally, we did not systematically assess participants' emotional state at the time of recording, nor did we control for potential variability in recording conditions. These unmeasured confounders represent important limitations, and future studies should incorporate such assessments to isolate the specific contributions of the primary condition under investigation. Fifth, the sample size, though sufficient for a multiverse exploration, remains modest for definitive correlate identification.

To advance this line of research, we recommend: (1) Cross cultural and multilingual validation of the identified acoustic features to examine their generalizability. (2) Integration of multimodal data—such as textual content, facial expressions, physiological signals (e.g., heart rate variability), and even neuroimaging or genetic correlates—to build more comprehensive predictive models. (3) Longitudinal studies that track acoustic changes alongside clinical trajectories, helping to establish causal and temporal links between vocal features and suicide ideation. (4) Development of practical screening tools that leverage robust acoustic features within user friendly, ethically designed platforms. This entails addressing technical challenges (e.g., real time feature extraction), ensuring data privacy, and embedding such tools within stepped care clinical workflows to support, not replace, human judgment.

## 5 Conclusion

This study demonstrates that while machine learning performance in detecting group differences based on suicidal ideation screening from speech is highly sensitive to analytical choices, a subset of acoustic features conveys stable, robust signals. By applying multiverse analysis, we have disentangled pipeline-dependent variance from consistent correlate candidates, underscoring the importance of methodological transparency in computational mental health. Because the groups were defined solely by screening measures and no independent assessments of depression, anxiety, or general distress were collected, the observed acoustic differences should be interpreted as associated with this composite group status rather than uniquely attributed to suicidal ideation or suicide risk. Future work should extend these findings to more diverse, longitudinal, and multimodal datasets, incorporating independent measures of related psychological constructs, with the ultimate goal of developing interpretable, ethically sound tools that can aid early risk detection and intervention in real-world settings.

## Data Availability

The data underpinning the findings of this study encompass sensitive personal information. Consequently, they are not publicly accessible owing to privacy and ethical constraints. De-identified data may be provided by the corresponding author upon a reasonable request and subject to appropriate ethical approval.
