# Supplementary material for: Multiverse analysis of machine learning: classification between groups defined by suicidal ideation screening status using acoustic features in college students
Source: Front Psychol. 2026 Jun 29;17:1785437. doi: 10.3389/fpsyg.2026.1785437 (PMC13358224; doi:10.3389/fpsyg.2026.1785437)
Supplement: Supplementary file 1 [file Supplementary_File_1.docx]

**Supplementary Materials**

**Supplementary Figures**

**
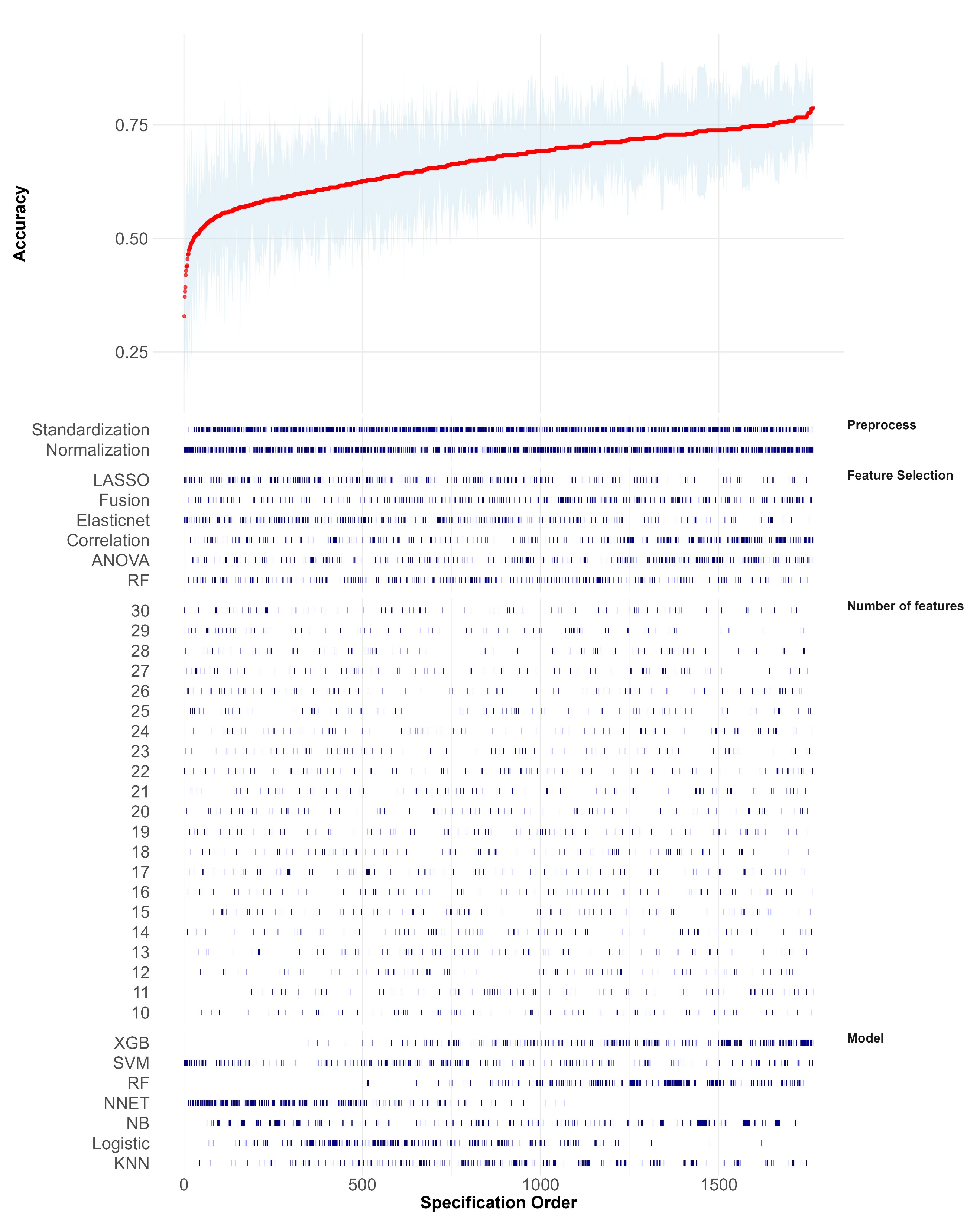
**

*Figure S1. Specification curve of Accuracy from the machine-learning multiverse analysis.*

**
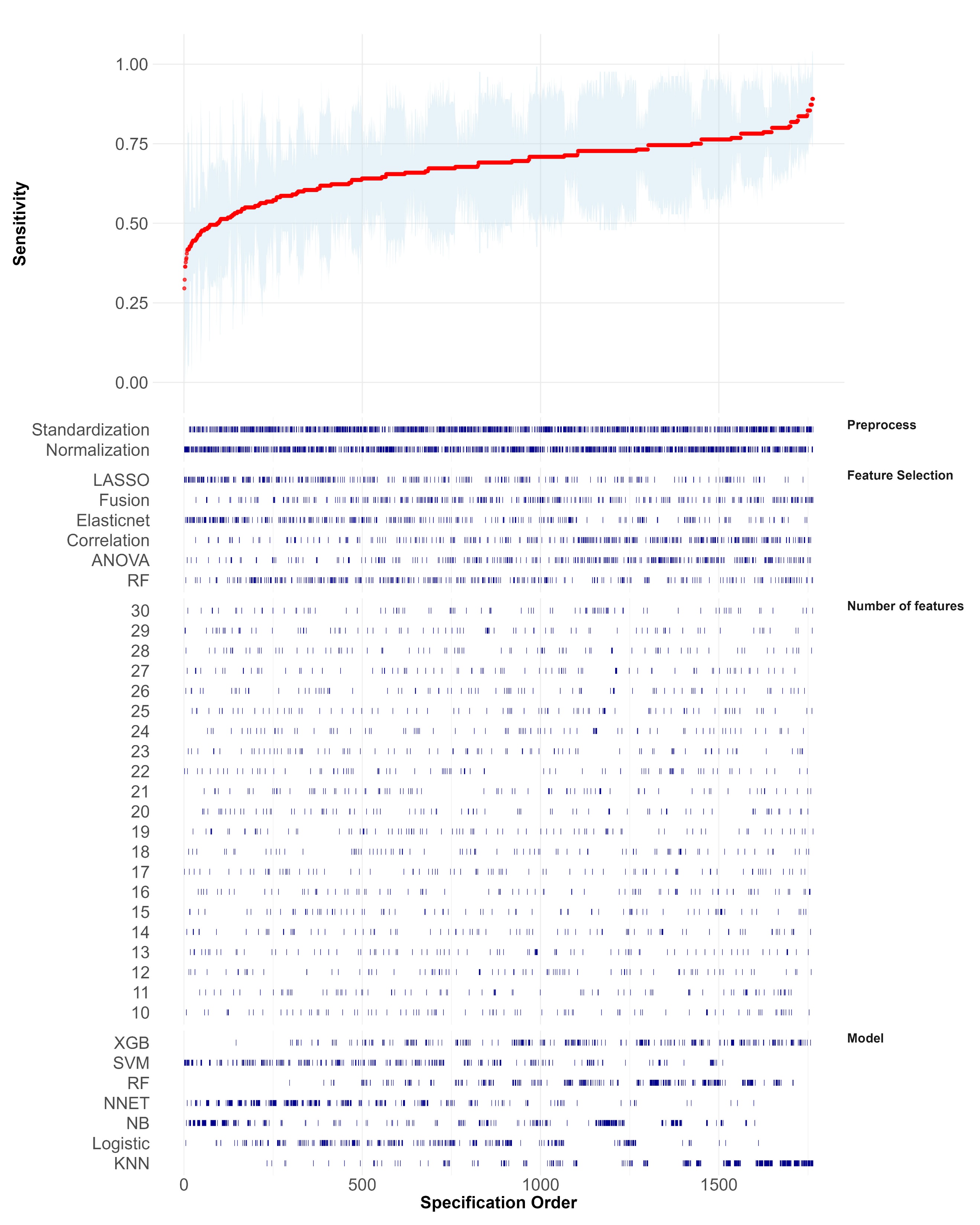
**

*Figure S2. Specification curve of Sensitivity from the machine-learning multiverse analysis.*

**
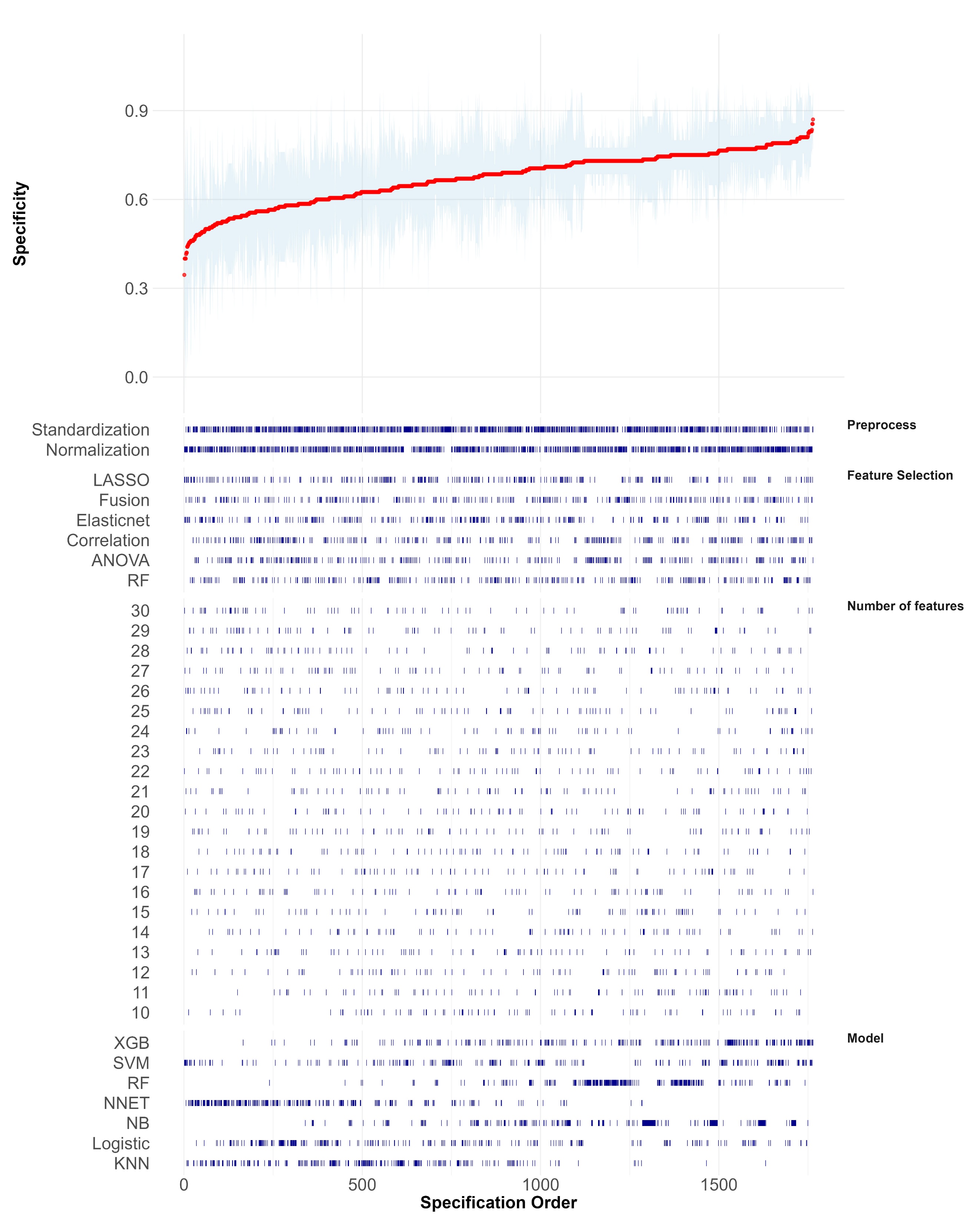
**

*Figure S3. Specification curve of Specificity from the machine-learning multiverse analysis.*


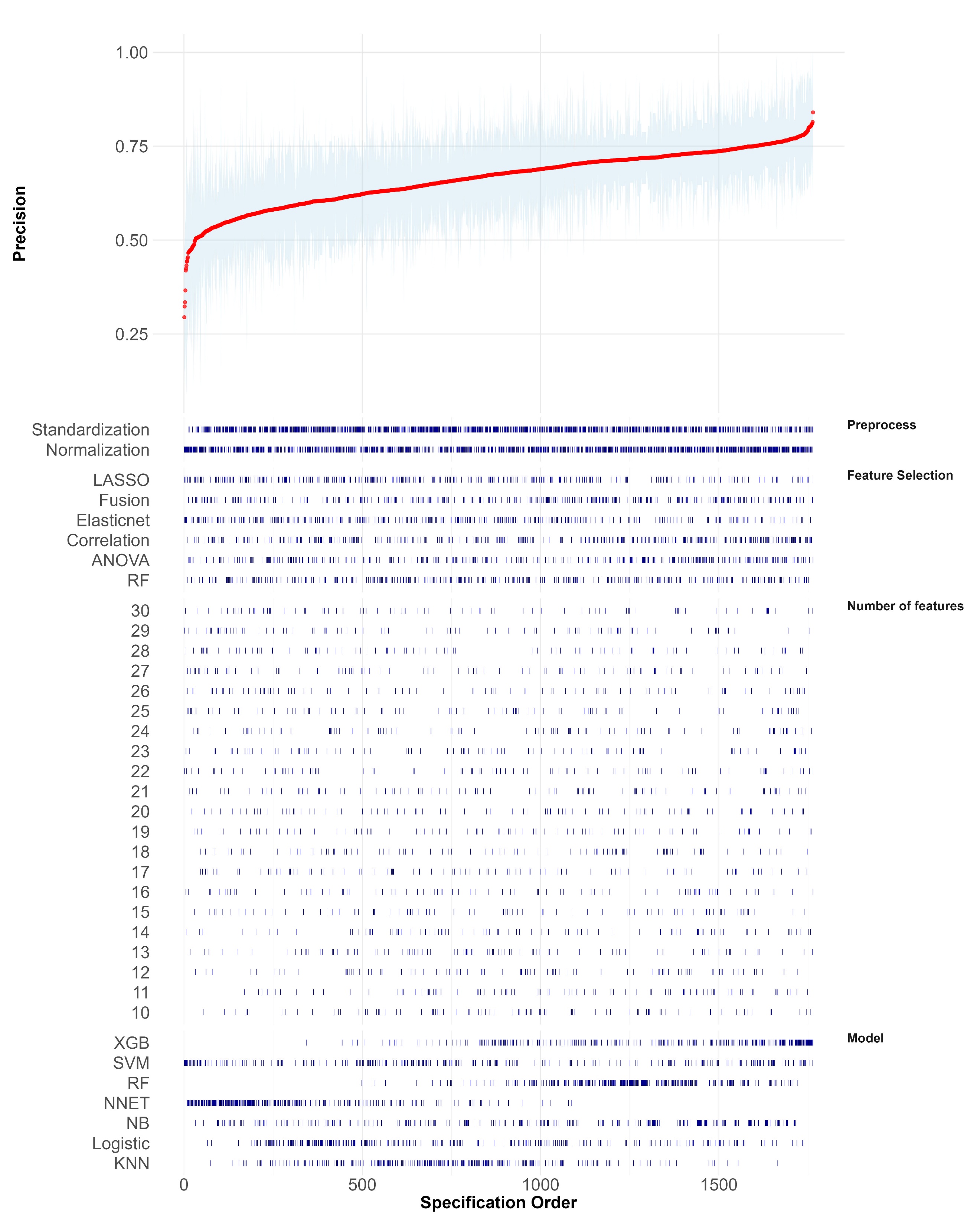


*Figure S4. Specification curve of Precision from the machine-learning multiverse analysis.*


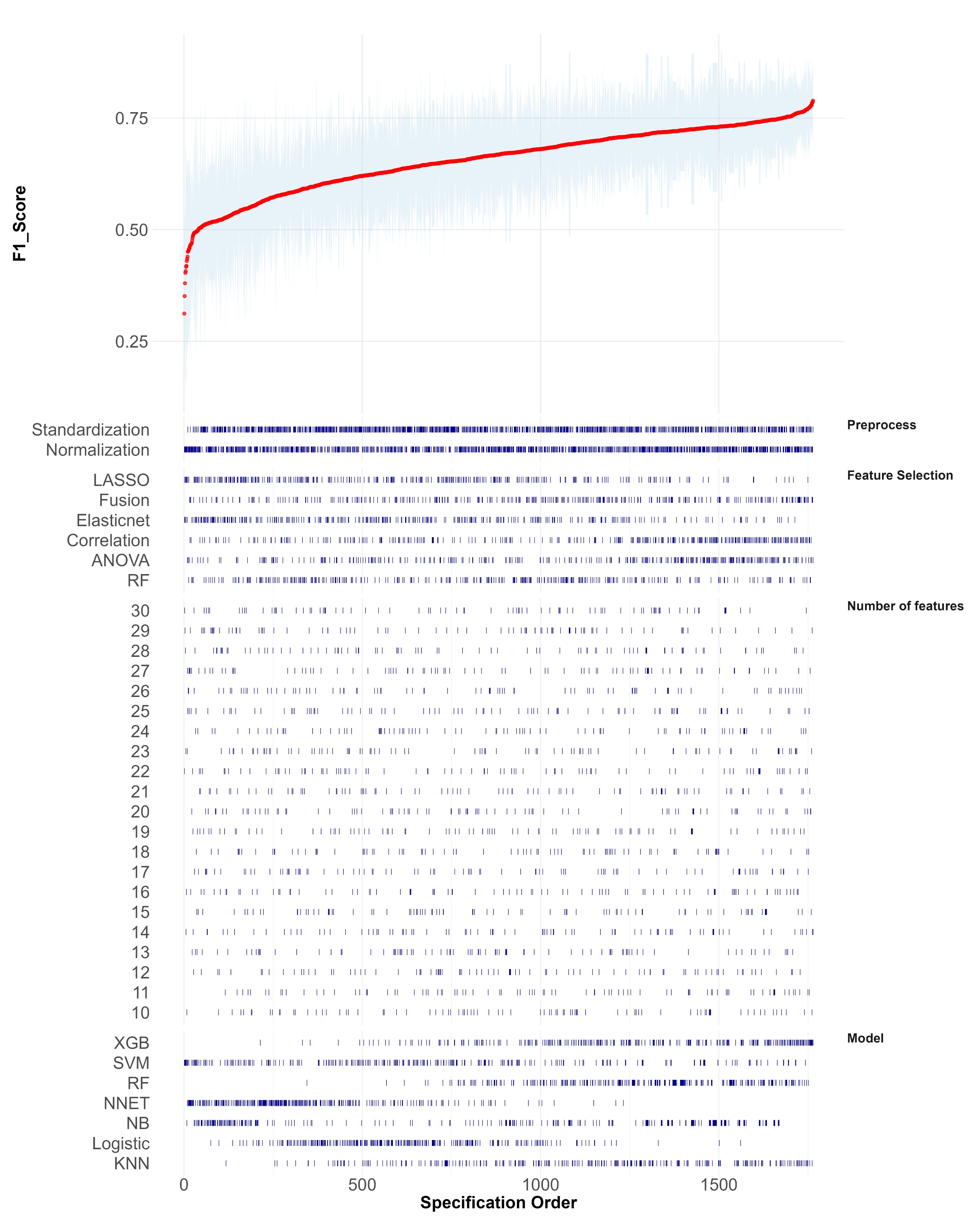


*Figure S5. Specification curve of F1-score from the machine-learning multiverse analysis.*

**Supplementary Tables**

Table S1. Overview of the Final Sentence

| **No.** | **Sentence** | **Sentence Mood** | **Sentence Function** | **Pleasure** | **Arousal** | **Dominance** | **Source** |
| --- | --- | --- | --- | --- | --- | --- | --- |
| 1 | “Do not impose on others what you yourself do not desire,” and “enter and depart as friends, watching over and assisting one another” — these reflect the “harmony” aspect of the core socialist values. | Declarative Sentence | Cognitive Function | 7.24 | 4.54 | 7.15 | Expert Evaluation |
| 2 | “The rise and fall of the nation is every citizen’s responsibility,” “a gentleman is broad‑minded while a petty person is anxious,” “one’s words must be kept and actions resolute,” and “the benevolent love others, the courteous respect others” — these proverbs exemplify the core socialist values of patriotism, dedication, integrity, and friendliness. |  |  | 7.37 | 4.74 | 7.02 |  |
| 3 | I despise my own family; it is one that saps one’s will to live(Shi, 2022). |  |  | 2.89 | 5.83 | 5.15 | Literature Source |
| 4 | I stood at a shabby podium and delivered a constrained speech with a face full of sorrow(Shi, 2022). |  |  | 2.43 | 6.15 | 4.35 |  |
| 5 | The grass is green.(Stasak et al., 2021) |  |  | 7.39 | 3.22 | 7.20 |  |
| 6 | On the glacier, the azure lake lay as calm and reflective as a mirror. |  |  | 7.59 | 3.33 | 7.26 | Expert Evaluation |
| 7 | Our country is making great strides toward prosperity, democracy, civility, and harmony—surely that is something to be proud of? | Rhetorical Question | Emotional Function | 7.63 | 5.70 | 6.83 |  |
| 8 | Why am I always so timid and helpless? |  |  | 2.81 | 6.44 | 4.26 |  |
| 9 | Hasn’t he always handled issues with steadfast composure? |  |  | 4.93 | 4.65 | 5.98 |  |
| 10 | What an exhilarating society of freedom, equality, justice, and rule of law! | Exclamatory Sentence |  | 7.30 | 5.19 | 6.76 |  |
| 11 | Everything feels so humiliating! |  |  | 2.70 | 6.52 | 4.78 |  |
| 12 | Tonight’s moonlight and breeze truly calm the spirit! |  |  | 7.85 | 3.81 | 7.52 |  |
| 13 | I truly wish that I were already dead.(Stasak et al., 2021) | Declarative Sentence | Suicide-related Expression |  | | | Literature Source |
| 14 | The world would be better off without me(Macdonald et al., 2024). |  |  |  |  |  |  |
| 15 | I felt an overwhelming sense of despair—what light remains in my life?(Macdonald et al., 2024) | Rhetorical Question |  |  |  |  |  |
| 16 | I feel like a giant sponge that has absorbed all the joy in the world, leaving only pain!(Macdonald et al., 2024) | Exclamatory Sentence |  |  |  |  |  |

Table S2. Sex-Stratified Analysis: Consistency of Effect Directions and Median Beta Estimates Across 240 Analytical Specifications

| Sex | Dimension | Choice | Count | Mean β | Min β | Median β | Max β |
| --- | --- | --- | --- | --- | --- | --- | --- |
| Male | Feature | F0_sma_iqr2-3 | 12 | 0.61 | 0.48 | 0.61 | 0.74 |
|  |  | F0_sma_stddev | 12 | 0.59 | 0.47 | 0.56 | 0.78 |
|  |  | F0env_sma_iqr1-2 | 12 | 0.57 | 0.42 | 0.53 | 0.78 |
|  |  | F0env_sma_linregc1 | 12 | 0.25 | 0.21 | 0.25 | 0.30 |
|  |  | F0env_sma_linregerrQ | 12 | 0.51 | 0.38 | 0.48 | 0.69 |
|  |  | F0env_sma_stddev | 12 | 0.60 | 0.44 | 0.55 | 0.85 |
|  |  | mfcc_sma[12]_kurtosis | 12 | 0.73 | 0.31 | 0.73 | 1.14 |
|  |  | mfcc_sma[2]_min | 12 | 0.40 | 0.38 | 0.40 | 0.42 |
|  |  | mfcc_sma[3]_kurtosis | 12 | 0.32 | 0.31 | 0.32 | 0.34 |
|  |  | mfcc_sma[5]_kurtosis | 12 | 0.57 | 0.40 | 0.56 | 0.75 |
|  | Process | Normalization | 40 | 0.51 | 0.21 | 0.46 | 1.14 |
|  |  | Raw | 40 | 0.51 | 0.21 | 0.46 | 1.14 |
|  |  | Standardization | 40 | 0.51 | 0.21 | 0.46 | 1.14 |
|  | Covariates | Age | 60 | 0.54 | 0.21 | 0.47 | 1.09 |
|  |  | None | 60 | 0.49 | 0.22 | 0.44 | 1.14 |
|  | Methods | Continuous_linear | 60 | 0.40 | 0.21 | 0.42 | 0.54 |
|  |  | Binary_logistic | 60 | 0.63 | 0.29 | 0.65 | 1.14 |
| Female | Feature | F0_sma_iqr2-3 | 12 | 0.21 | 0.14 | 0.22 | 0.28 |
|  |  | F0_sma_stddev | 12 | 0.17 | 0.09 | 0.18 | 0.24 |
|  |  | F0env_sma_iqr1-2 | 12 | 0.22 | 0.17 | 0.22 | 0.26 |
|  |  | F0env_sma_linregc1 | 12 | 0.29 | 0.25 | 0.29 | 0.33 |
|  |  | F0env_sma_linregerrQ | 12 | 0.22 | 0.16 | 0.23 | 0.25 |
|  |  | F0env_sma_stddev | 12 | 0.30 | 0.22 | 0.32 | 0.35 |
|  |  | mfcc_sma[12]_kurtosis | 12 | 0.13 | 0.05 | 0.11 | 0.22 |
|  |  | mfcc_sma[2]_min | 12 | 0.14 | 0.02 | 0.14 | 0.25 |
|  |  | mfcc_sma[3]_kurtosis | 12 | 0.25 | 0.16 | 0.26 | 0.33 |
|  |  | mfcc_sma[5]_kurtosis | 12 | 0.12 | 0.09 | 0.12 | 0.16 |
|  | Process | Normalization | 40 | 0.21 | 0.02 | 0.22 | 0.35 |
|  |  | Raw | 40 | 0.21 | 0.02 | 0.22 | 0.35 |
|  |  | Standardization | 40 | 0.21 | 0.02 | 0.22 | 0.35 |
|  | Covariates | Age | 60 | 0.18 | 0.02 | 0.20 | 0.33 |
|  |  | None | 60 | 0.23 | 0.05 | 0.24 | 0.35 |
|  | Methods | Continuous_linear | 60 | 0.19 | 0.05 | 0.19 | 0.33 |
|  |  | Binary_logistic | 60 | 0.22 | 0.02 | 0.23 | 0.35 |

**Supplementary Data**
